# Shallow periorbital injection of triamcinolone acetonide in treatment of lower eyelid entropion related to thyroid-associated ophthalmopathy: Three case reports

**DOI:** 10.1097/MD.0000000000019026

**Published:** 2020-01-31

**Authors:** Fei Mo, Dongdong Xu, Haiyan Xu, Gang Li, Yizhuo Gao, Hui Li

**Affiliations:** a Department of Ophthalmology, Peking Union Medical College Hospital, Chinese Academy of Medical Sciences, Beijing; b Department of Ophthalmology, Xi’an 4th Hospital, Xi’an, China.

**Keywords:** entropion, periorbital injection, thyroid-associated ophthalmopathy, triamcinolone acetonide

## Abstract

**Introduction:**

Entropion and secondary trichiasis can lead to irritative symptoms and essential damage of ocular surface. There is no literature reporting the lower eyelid entropion related to thyroid-associated ophthalmopathy (TAO), let alone the treatment. Treatment based on etiology may yield effective and sustained results. We report 3 case reports of lower eyelid entropion associated with TAO, and provide an effective and persistent alternative to cure this entropion via the administration of shallow periorbital injections of triamcinolone acetonide (TA).

**Patient concerns:**

Three patients presented irritative symptoms of ocular surface and diplopia.

**Diagnosis:**

According to thyroid dysfunction, physical examination, and imaging findings of extraocular muscle involvement, TAO and unilateral or bilateral lower eyelid entropion were diagnosed.

**Interventions:**

We administered shallow periorbital injections of TA to the affected eye at 3- to 4-week intervals depending on clinical response.

**Outcomes:**

All patients underwent complete correction of the lower eyelid entropion and no recurrence was found.

**Conclusion:**

The cause of lower eyelid entropion related to TAO might be the immunoinflammatory reaction of the lower eyelid retractors, enhancing the traction of pulling the lower eyelid inferoposteriorly. This condition can be treated with shallow periorbital injections of TA. Histopathological evidence and randomized controlled trials are expected to confirm our hypothesis.

## 1 Introduction

Entropion is a common eyelid malposition in which the margin of the eyelid turns inward against the globe. Often, the eyelashes are also directed posteriorly. The most recognized classifications of entropion are congenital, involutional, cicatricial, and spastic.^[1]^ Currently, no publication has presented entropion related to thyroid-associated ophthalmopathy (TAO). And, previous studies have confirmed that TAO can cause immunoinflammatory changes in the levator and Müller muscles.^[2,3]^ We thus believe the cause of entropion with TAO is the immunoinflammatory reaction of the lower eyelid retractors, although we have no histopathologic evidence to support this theory. The lower eyelid retractors, one of the posterior lamella components of the eyelid, consist of the capsulopalpebral fascia and the inferior tarsal muscle that pull the lower eyelid inferoposteriorly.^[4,5]^ When inflammation or fibrosis infiltrates in the lower eyelid retractors, breaking the balance of eyelid movements mediated by the orbicularis oculi and the levator palpebrae muscles, entropion and secondary trichiasis of the lower eyelid will occur. These changes can lead to ocular surface irritation and substantial corneal injuries such as superficial punctate keratopathy, corneal abrasion, infection, vascularization, opacities, and loss of vision.^[6]^ Hence, it is necessary to reverse the ectopic eyelid. Strategies for entropion consist of both nonsurgical and surgical procedures. Medical treatments such as applying tissue glue and botulinum toxin A can be used for the temporary management of entropion.^[7,8]^ Surgical correction can present complications.^[9]^ Considering the immunoinflammatory reaction of the lower eyelid retractors, we began to use triamcinolone acetonide (TA), a long-term glucocorticoid, as an alternative option. We report our results here. To the best of our knowledge, this is the first article discussing the treatment of lower eyelid entropion related to TAO with TA.

## 2 Methods

In our technique, the lower eyelid is sterilized with 5% povidone-iodine. A total volume of 0.5 mL of TA (40 mg/mL; Kunming Jida Pharmaceutical Co., Ltd.) is injected using a 25-gauge, 0.5-in. needle. The needle is introduced percutaneously about 1 cm inferior to the margin of the lower eyelid from the infranasal or infratemporal side, where the inversion is more apparent. The injection is then delivered, and an eye pad is taped over the eyelid for ∼10 min. Injections are repeated at 3- to 4-week intervals depending on clinical response. We recorded improvements in the patients’ irritation symptoms and observed the direction of the eyelashes before and after the injection, and whether the eyelashes continued to contact the cornea or conjunctiva. All the patients received the full course (Table 1).

**Table 1:**
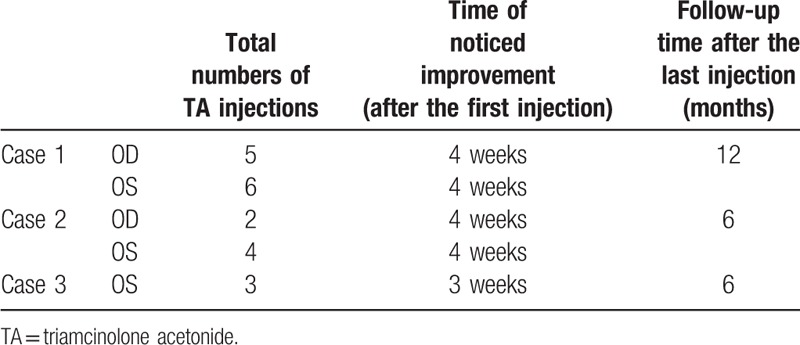
Patient summary.

### 2.1 Case 1

**Figure 1:**
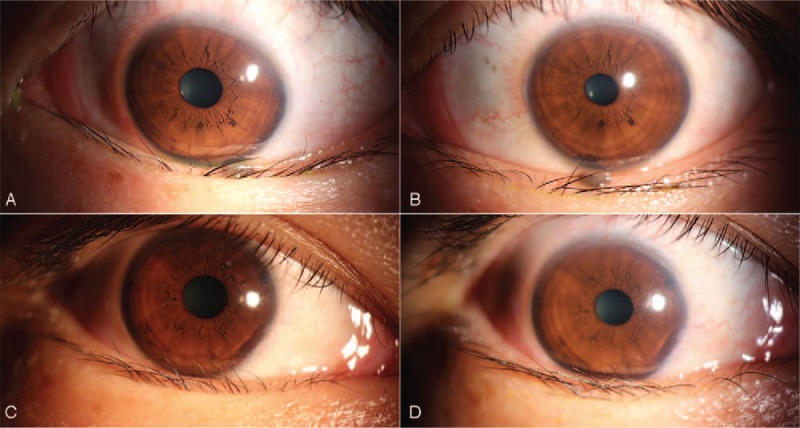
(A) Pre-treatment of the left eye. (B) Post-treatment of the left eye. (C) Pre-treatment of the right eye. (D) Post-treatment of the right eye.

A 35-year-old female was suffering from lower eyelid entropion and trichiasis in her left eye accompanied by other ocular abnormalities when she first came to our clinic. She had previously undergone iodine-131 treatment due to hyperthyroidism and then, 2 years ago, acquired secondary hypothyroidism. From then on, she supplemented with L-thyroxine. Although conjunctival congestion and restrictive hypotropia in her left eye were also observed, there were no significant findings in her visual acuity, intraocular pressure, residual anterior segment, or fundus examination. Eventually, she was diagnosed with TAO and treated with diminishing prednisolone orally for 3 months. During this period, the degree of hypotropia reduced considerably, but the inverted eyelid did not resolve. Meanwhile, her lower right eyelid began to turn inward. We began administering shallow periorbital injections of TA to both eyes monthly for 6 months. The inverted lower eyelid and eyelashes gradually returned to normal. There was no recurrence at 1 year post treatment (Fig. 1).

### 2.2 Case 2

**Figure 2:**
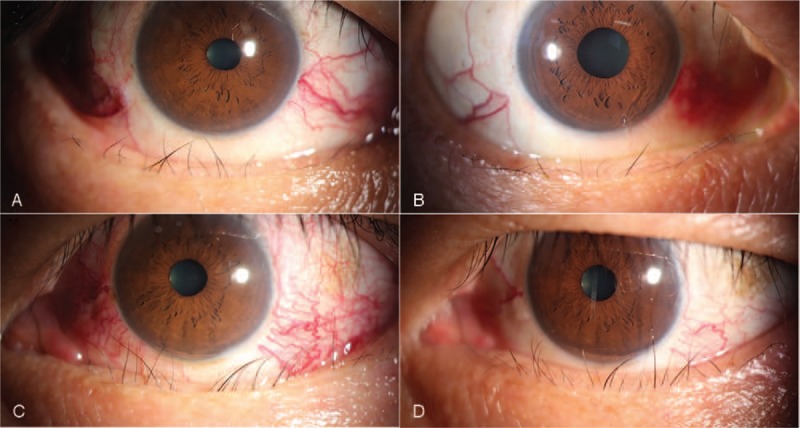
(A) Pre-treatment of the right eye. (B) Post-treatment of the right eye. (C) Pre-treatment of the left eye. (D) Post-treatment of the left eye.

A 47-year-old male presented with a 16-month history of hyperthyroidism and TAO. He had been treated multiple times with intravenous methylprednisolone. He complained of photophobia, tearing, proptosis, and diplopia when he came to our clinic. Slit lamp biomicroscopy showed entropion and misdirected cilia in both lower eyelids and local corneal abrasion in his left eye. The patient presented with obvious engorged sclera vessels and caruncle congestion in both eyes. He also showed esotropia at 5 m distance and restrictive abduction. We began injecting TA monthly in the shallow periorbital areas of his eyes for a total of two injections in the right eye and four in the left eye. It is worth mentioning that the intraocular pressure of both eyes increased after the first injection, but this was able to be controlled by medications. At the end of the first month, both inverted lower lids had turned outward. At 2 months post-treatment, the misdirected cilia in the right eye had recovered completely. Despite another two injections, however, he began to experience refractory trichiasis in his left eye. He eventually underwent strabismus surgery to eliminate diplopia. The patient was followed for 6 months after the last injection, and no recurrence of entropion was observed (Fig. 2).

### 2.3 Case 3

**Figure 3:**
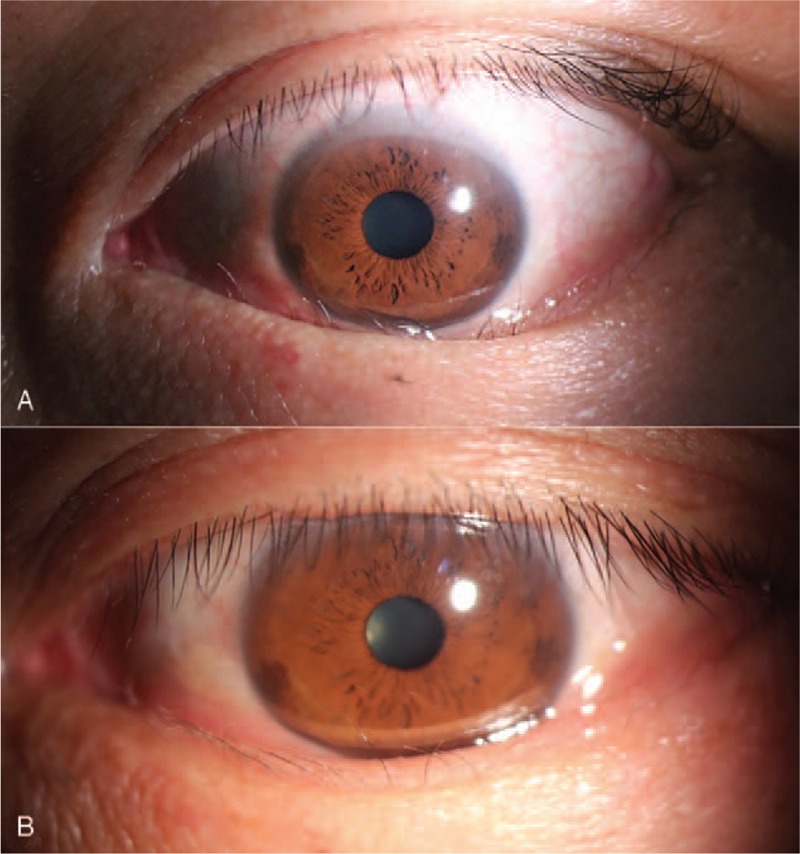
(A) Pre-treatment. (B) Post-treatment.

A 47-year-old male complained of a foreign body sensation, tearing, and discharge in his left eye. He had been diagnosed with subclinical hyperthyroidism and TAO. He was treated with propylthiouracil, L-thyroxine, and reduction of prednisolone at the same time. Clinical examination revealed restricted eye movement and hypotropia in his left eye. Slit lamp examination presented unilateral left entropion and acquired trichiasis of the lower eyelid. He also showed mild chemosis and conjunctival congestion. Visual acuity for both eyes was 1.0 (20/20). The patient then received TA injection in the shallow periorbital areas of his left eye. There was remarkable improvement in the patient's entropion and trichiasis in the third week of treatment. His inverted lower eyelid had completely everted to the normal eyelid margin with a few residual inverted eyelashes. However, there was not obvious alleviation of strabismus angle. Then, he was given two more TA injections. His irritative symptoms gradually disappeared. Later, he underwent strabismus surgery to alleviate his diplopia. At 6-month follow-up, the patient had no recurrence of trichiasis or entropion (Fig. 3).

## 3 Discussion

This is the first article illustrating a new technique to treat lower lid entropion related to TAO. In our study, we speculated that entropion with TAO was caused by the immunoinflammatory reaction of the lower eyelid retractors. The successful treatment of this condition via the administration of shallow periorbital injections of TA to the affected eye confirmed this hypothesis. However, we still lack essential histopathological evidence.

The location of periorbital injection is determined by the anatomical position of the lower eyelid retractors. The average lower tarsal plate measures 4 mm vertically, and the orbital septum fuses with the capsulopalpebral fascia 5 mm inferior to the tarsus.^[4]^ By adding the two figures, we determined that the percutaneous introduction of the needle about 1 cm inferior to the margin of the lower eyelid was sufficient to target the lower lid retractors.

Glucocorticoids have become well known for their immunosuppressive and anti-inflammatory effects.^[10]^ Many studies have also demonstrated that TA, a synthetic glucocorticoid, can effectively reduce inflammation of the levator palpebrae superioris muscle and Müller's muscle in patients with TAO.^[11,12]^ Moreover, Lee et al hypothesized that subconjunctival injection of TA may induce atrophy of the Müller muscle.^[13]^ The primary components of lower eyelid retractors are the capsulopalpebral fascia and the inferior tarsal muscle, which are respectively analogous to the levator aponeurosis of the upper lid and the Müller muscle. Therefore, it is rationale to speculate that TA can reduce inflammation of the lower eyelid retractors and weaken their inferoposterior tractional force and improve entropion further by alleviating muscle edema and preventing fibrosis.

The patients in Cases 1 and 3 presented with active TAO on acceptance into our study. Their signs of entropion did not improve during treatment with systemic steroid therapy until we began local injection of TA. Insufficient drug concentrations at the location of inflammation may be the reason. Furthermore, the reduction of systemic glucocorticoid and continued inflammatory activity may account for the occurrence of contralateral lower lid entropion in the Case 1 patient.

After multiple TA injections, the patient in Case 2 achieved bilateral recovery of entropion but continued to present with misdirected cilia in the left eye. In TAO, the active phase of the condition usually lasts about 1 year before the onset of the stationary phase.^[14]^ The course of Case 2 had lasted 16 months and his ocular condition was under a relatively static phase when he began receiving treatment. And the signs of entropion and trichiasis were more prominent in his left eye. This may explain why intractable trichiasis of the patient's left eye did not respond to the steroid.

Local complications of steroid injection around the eye include globe perforation, intractable elevated intraocular pressure, conjunctival or corneoscleral melting, and vascular occlusion from embolization.^[15]^ Among our cases, the only side effect of shallow periorbital injection of TA was a transient increase in the patient's intraocular pressure in Case 2, and this was able to be controlled with topical medications. And all three patients showed only slight redness around the injection point, without blurring, bleeding, infection, etc.

In our cases, all patients improved significantly at the 1 month of the treatment. The longest follow-up was 1 year, and no recurrence was found. And patients with active inflammation were more sensitive to the steroid. We thus propose that patients who undergo treatment during the active phase are more likely to achieve complete resolution.

In summary, this case series proposes a characteristic disorder, lower eyelid entropion associated with TAO, that can be corrected by the shallow periorbital injection of TA. We provide an effective and persistent alternative to cure entropion that is extremely suitable for patients who reject surgery. Histopathological research and randomized controlled trials are expected to confirm our conclusion.

## Acknowledgments

The authors thank the patients participating in the present study who provided written permission for publication of this case report.

## Author contributions

**Data curation:** Haiyan Xu, Gang Li, Yizhuo Gao.

**Investigation:** Fei Mo, Dongdong Xu.

**Methodology:** Fei Mo, Dongdong Xu.

**Project administration:** Hui Li.

**Supervision:** Hui Li.

**Validation:** Hui Li.

**Writing – original draft:** Fei Mo, Dongdong Xu.

**Writing – review & editing:** Fei Mo, Dongdong Xu, Haiyan Xu, Gang Li, Yizhuo Gao, Hui Li.
